# *Ghd8* controls rice photoperiod sensitivity by forming a complex that interacts with *Ghd7*

**DOI:** 10.1186/s12870-019-2053-y

**Published:** 2019-11-01

**Authors:** Peng Wang, Rong Gong, Ying Yang, Sibin Yu

**Affiliations:** 1 National Key Laboratory of Crop Genetic Improvement, College of Plant Science and Technology, Huazhong Agricultural University, Wuhan, China; 2 Present Address: Department of Agronomy and Horticulture, University of Nebraska Lincoln, Lincoln, NE USA

**Keywords:** Photoperiod sensitivity, Flowering time, Molecular interaction, CCAAT-box motif, Rice

## Abstract

**Background:**

Flowering time is one of the most important agronomic characteristics that ultimately determine yield potential and eco-geographical adaptation in crops. *Ghd8* and *Ghd7*, two major flowering genes, have similar functions and large pleiotropic effects in controlling the heading date, plant height and grain yield of rice. However, these gene interactions at the genetic and molecular levels have not been determined to date.

**Results:**

In this study, we investigated the genetic interaction between *Ghd8* and *Ghd7* by using a set of near-isogenic lines and a panel of natural germplasm accessions in rice. We found that *Ghd8* affected multiple agronomic traits in a functional *Ghd7*-dependent manner*.* Both functional *Ghd8* and *Ghd7* are pivotal for rice photoperiod sensitivity controlled by *Hd1* and *Hd3a*. GHD8 could form a heterotrimeric complex with HD1 and OsHAP5b to activate the transcription of *Ghd7* by binding directly to the promoter region of *Ghd7*, which contains the CCAAT-box motif.

**Conclusions:**

The results of this study help to elucidate the genetic and molecular bases of *Ghd8* and *Ghd7* interactions, indicating that *Ghd8* acts upstream of *Ghd7* to activate its transcription, which inhibits *Hd3a* expression and thus affects flowering time and rice adaptation.

## Background

Photoperiod sensitivity (PS) is defined as the developmental responses of plants to the relative lengths of light and dark periods and confers on many plant species the ability to adapt to a range of growing season periods by means of adjusting flowering time. Flowering time or heading date (HD) is one of the most important agronomic traits in crops. The probable initiation of flowering time in response to climate change largely determines the crop yield potential [1]. This property is observed because a positive correlation was found between grain yield and flowering time in various natural conditions [2], and either too early or too late flowering might cause reduced grain yield. It has been reported that the photoperiod insensitivity in crops due to loss-of-function mutants or weak alleles of flowering time genes restricts their distributions at specific environmental conditions, such as high latitudes [3–6].

The regulatory mechanisms of flowering time have been extensively studied in *Arabidopsis* and rice [7, 8]. As a short-day plant, rice (*Oryza sativa* L.) can flower promptly under short-day (SD) conditions and flower relatively late under long-day (LD) conditions. Two independent gene pathways have been reported to be involved in regulating flowering time under both conditions. The OsGI-Hd1-Hd3a (rice GIGANTEA, Heading date 1 and Heading date 3a) signaling pathway in rice is evolutionarily conserved as the GI-CO-FT (GIGANTEA, CONSTANS, and FLOWERING LOCUS T) pathway in *Arabidopsis*. Among these genes, the expression of florigen genes in the downstream pathway, such as *Hd3a/FT* plays central roles in determining flowering time. High expression of *Hd3a/FT* strongly accelerates flower time, and downregulation of its expression delays or prevents flowering [9, 10]. In recent years, several novel flowering genes have been identified in rice; they have no orthologs in the *Arabidopsis* genome and constitute a rice-specific flowering pathway. For example, *Ghd7* (Grain number, plant height and heading date 7), a homolog group of CO, CO-like, and TOC1 (CCT)-domain proteins, was identified as a repressor of flowering through inhibiting *Hd3a* under LD conditions [11]. *Ehd1* (Early heading date 1) was identified as the rice-specific flowering signal integrator and acts upstream of *Hd3a* [12]. In addition, several flowering time genes have recently been identified to participate in either of the two main independent signaling pathways or even link them. *Ghd7.1*/*OsPRR37* harboring a conserved CCT domain was reported to inhibit *Ehd1* and *Hd3a* only under LD conditions but was independent of *Hd1*. *Ghd8/DTH8* (Grain number, plant height and heading date 8), encoding a CCAAT-box binding factor, known as a HAP3/NF-YB protein, was identified as a major effect locus affecting flowering with the dual function to inhibit flowering under LD conditions and promote flowering under SD conditions by regulating *Ehd1* and *Hd3a*. Moreover, GHD8, HD1 and OsHAP5/NF-YC subunits could form a heterotrimeric complex to bind the CORE element at the promoter of *Hd3a* to directly regulate its expression [6]. *Hd1* is a gene that was reported to genetically interact with other flowering time genes, such as *Hd2/PRR37, Ghd8* (*DTH8/LHD1/Hd5/LH8*), *Hd6*, *SE5* and *Ghd7* [13–23]. Some of these interactions were further validated at the molecular level, showing a complex of protein-protein interactions to regulate the expression of downstream genes. For example, HD1 and GHD7 proteins form a complex to specifically bind to a *cis*-regulatory region in *Ehd1* and repress its expression [24, 25]. The revelation of interaction among genes at the genetic and molecular levels has considerably enhanced our understanding of the regulatory networks for flowering time.

*Ghd7* and *Ghd8* are two major genes identified recently with the same pleiotropic effect on the number of grains, plant height and heading date in rice. Previous results showed that a strong genetic interaction exists between *Ghd7* and *Ghd8*, which seriously inhibits flowering time in the rice natural population [26]. The interaction effect could be strongly enlarged by a functional *Hd1*. However, it remains unknown whether and how these two genes *Ghd7* and *Ghd8* interact at the molecular level. To address this question, a set of near-isogenic lines (NIL) and a rice core collection panel were first used to investigate the genetic interaction effect of *Ghd7* and *Ghd8.* Then, transcription analysis, electrophoretic mobility shift assay (EMSA) and chromatin immunoprecipitation (ChIP) assays were conducted for a likely molecular interaction. Our results revealed that *Ghd8* induces the transcription of *Ghd7* via GHD8-OsHAP5b-HD1 complex binding to the specific CCAAT-box region in the *Ghd7* promoter. Under both SD and LD conditions, *Ghd8* might form a complex with OsHAP5B and HD1 activates the transcription of *Ghd7* to inhibit the expression of *Ehd1* and *Hd3a*, leading to late flowering. Both functional *Ghd8* and *Ghd7* are pivotal for rice PS controlled by *Hd1* and *Hd3a*. These results regarding molecular and genetic interactions provide new insights into the gene-regulatory networks controlling flowering time and adaptation in rice.

## Results

### *Ghd8* acts on agronomic traits depending on functional *Ghd7*

**Fig. 1:**
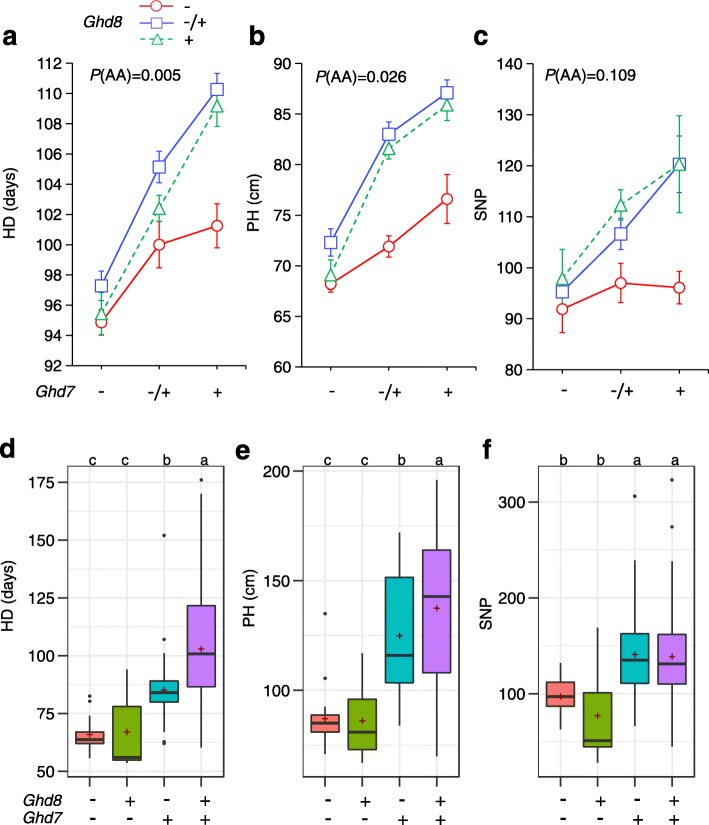
Genetic interaction of *Ghd7* and *Ghd8* contributes to the variation of HD, PH and yield in rice. Heading date (HD, a), plant height (PH, b) and spikelet number per panicle (SNP, c) were scored in the near-isogenic line (NIL)-derived population under natural SD conditions in Hainan (N18.48, E110.02). The open red circle, blue square, and green triangle indicate nonfunctional *ghd8*, heterozygous, and functional *Ghd8*, respectively. HD(d), PH(e), and SNP (f) in four haplotypes of *Ghd8* and *Ghd7* in the core collection (196 accessions) grown under natural LD conditions in Wuhan (N30.52, E114.3). The different letters above the boxplots represent significance among the haplotypes at *p* < 0.05 by Tukey’s HSD test. *p* values indicate the significance of the additive interaction effect between *Ghd7* and *Ghd8*

Both *Ghd8* and *Ghd7* have been identified as major effect loci, exhibiting pleiotropy in affecting heading date (HD), plant height (PH) and grain yield in rice [11, 27]. The significant interaction also affected heading date [26]. To better understand how this interaction acts on different agronomic traits, we developed an F_2_ population derived from a cross between near-isogenic lines NIL-Ghd8 and NIL-Ghd7 within the common Zhenshan 97B (ZS97) background. The genetic analyses showed that *Ghd8* and *Ghd7* interact significantly to affect HD (*p* = 0.005) and PH (*p* = 0.026) in the segregating population (Fig. 1a and b). Significant difference (*p* < 0.001) in the three traits HD, PH and spikelet number per panicle (SNP) was detected among the three genotypes at *Ghd8* within the functional *Ghd7* background (Fig. 1a–c). However, no significant difference was found in the assayed traits within the nonfunctional *ghd7* background.

The significant interaction of *Ghd8* and *Ghd7* was also revealed in a core collection panel consisting of 196 rice varieties. The Tukey honest test (α = 0.05) showed that the varieties carrying *Ghd8ghd7* (only *Ghd8* is functional) were not significantly different in HD, PH and SNP compared with the varieties carrying *ghd8ghd7* (both alleles are loss of function) under natural LD conditions (Fig. 1d–f). However, the varieties carrying *Ghd8Ghd7* showed significantly delayed HD and increased PH and SNP compared with the *ghd8ghd7* and *Ghd8ghd7* varieties. In particular, the varieties carrying *Ghd8Ghd7* exhibited a significant delay in HD and an increase in PH compared with *ghd8Ghd7*. These results indicate that *Ghd8* affected agronomic traits in a functional *Ghd7-*dependent manner. Hence, the results confirmed that *Ghd8* genetically interacts with *Ghd7* delaying flowering time and increasing plant height in both homogeneous and heterogeneous backgrounds.

### Interaction of *Ghd8* and *Ghd7* is pivotal for *Hd1* and *Hd3a* to control PS

**Fig. 2:**
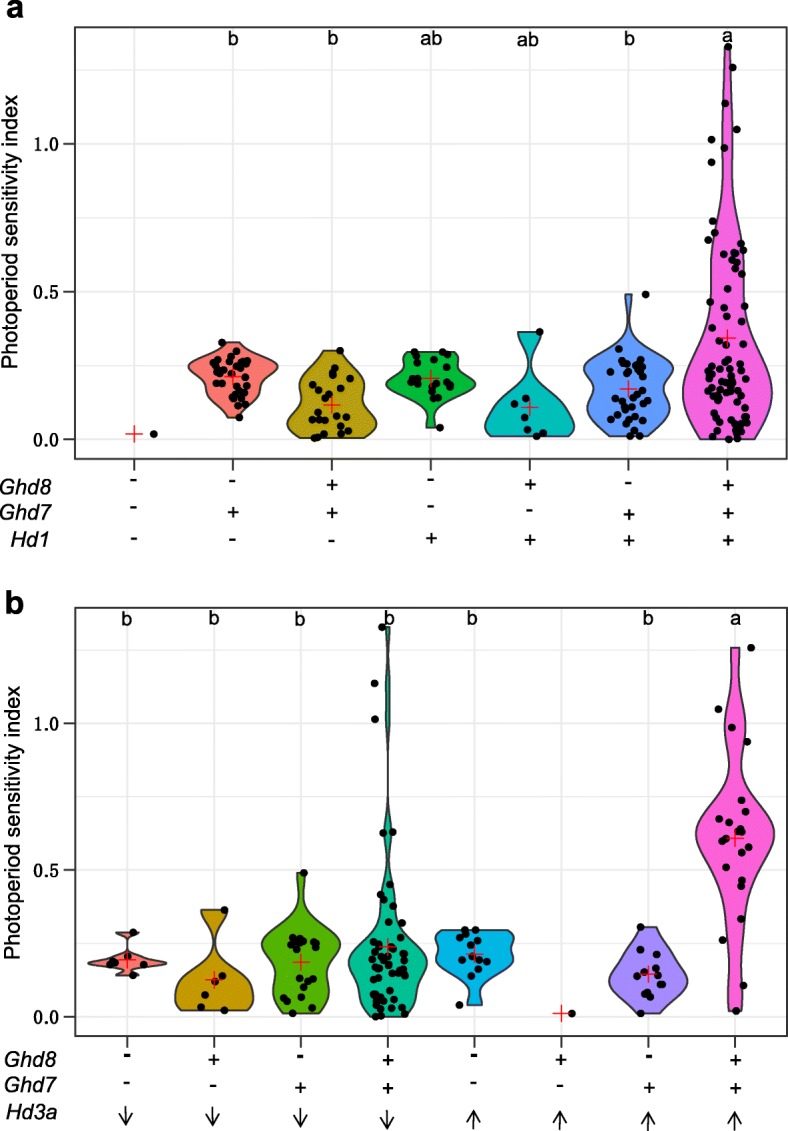
Genetic interaction between *Ghd7* and *Ghd8* conditioned *Hd1* and *Hd3a* affecting PS in rice. (a) Comparison of photoperiod sensitivity (PS) index among seven genotype types classed by three functional genes (*Ghd7*, *Ghd8*, *Hd1*) in 196 varieties as shown by the violin box. *Ghd8ghd7hd1* was not available in the core collection. (b) Comparison of PS among eight genotype types classed by two genes (*Ghd7*, *Ghd8*) and the *Hd3a* promoter in 139 out of the 196 varieties. The promoter types of *Hd3a* were selected based on the previous report [31]. The up and down arrows indicate the high and low expression levels of the *Hd3a* types. Fifty-seven varieties with nonfunctional *hd1* were not included in the (b) analysis. The red plus symbol indicates the average of PS. For *Ghd8*, *Ghd7* and *Hd1* genes, the “+” and “-” symbols at the bottom of the plots indicate the functional and nonfunctional alleles. The different letters on the top of the plots indicate the significant differences in PS among haplotypes by Tukey’s HSD test (α = 0.05). Because there was only one variety, haplotypes *ghd8ghd7hd1* and *Ghd8ghd7Hd3a* (high expression) were not used in the analysis

Photoperiod sensitivity is an important factor of rice cultivars to adapt to variable environments. It has been reported that *Hd1* is a key gene that largely determines rice PS [28]. Given that both *Ghd7* and *Ghd8* are involved in the *Hd1-Hd3a* regulatory pathway [25, 29, 30], we tested whether the interaction between *Ghd7* and *Ghd8* or *Ghd7* and *Ghd8* alone can affect the function of *Hd1* on PS. The core collection was planted under both LD and SD conditions. The PS index of each variety was calculated by the formula (|HD^LD^- HD^SD^|)/ HD^LD^ (Additional file 1: Table S1). A large variation in PS was observed in the rice core collection. *Ghd8Ghd7Hd1* (all are functional alleles) revealed significantly higher PS on average than *Ghd8Ghd7hd1* (*hd1* is nonfunctional) (Fig. 2a), suggesting that *Hd1* is the key gene determining PS variation, as reported before [28]. Haplotype *Ghd8Ghd7Hd1* also showed significantly higher PS than haplotype *ghd8Ghd7Hd1*, and the latter revealed a slightly higher PS that was not significant than haplotypes *Ghd8ghd7Hd1* and *ghd8ghd7Hd1*. These results indicate that functional *Ghd8* and *Ghd7* and their interaction were required for the function of *Hd1* on PS. In addition, *Hd3a*, the other gene determining the PS of rice, also showed a similar pattern (Fig. 2b), suggesting that both functional *Ghd8* and *Ghd7* and their interaction played an important role in affecting the expression level of *Hd3a* on the control of PS. The varieties carrying functional *Ghd8*, *Ghd7*, and *Hd3a* promoter belonging to the high expression type [31] revealed a significantly higher level in the average PS than the other haplotypes. However, this significance was not observed in the varieties carrying functional *Ghd8* and *Ghd7* along with the promoter of *Hd3a* belonging to the low expression type.

In addition, a segregating population segregated at three loci, *Ghd7*, *Ghd8* and *Hd1,* within the homogeneous background of ZS97 was used to analyze the effects of interaction among *Ghd8*, *Ghd7* and *Hd1*. The analysis of variance among genotypes showed that under natural LD conditions, the functional *Hd1* (ZS97 allele) showed the strongest effect to delay HD (average = 160 d) in that genotypes carrying the *Ghd8Ghd7Hd1* allele combination when both *Ghd7* (9311 allele) and *Ghd8* (9311 allele) were functional; however, the *ghd8Ghd7Hd1* or *Ghd8ghd7Hd1* genotypes carrying either single functional *Ghd7* or *Ghd8* exhibited earlier heading dates (Additional file 3: Figure S1). These results further indicate that the interaction between *Ghd8* and *Ghd7* dramatically increased the *Hd1* effect on the control of heading date in rice.

### *Ghd8* upregulates *Ghd7* expression to inhibit *Hd3a*

**Fig. 3:**
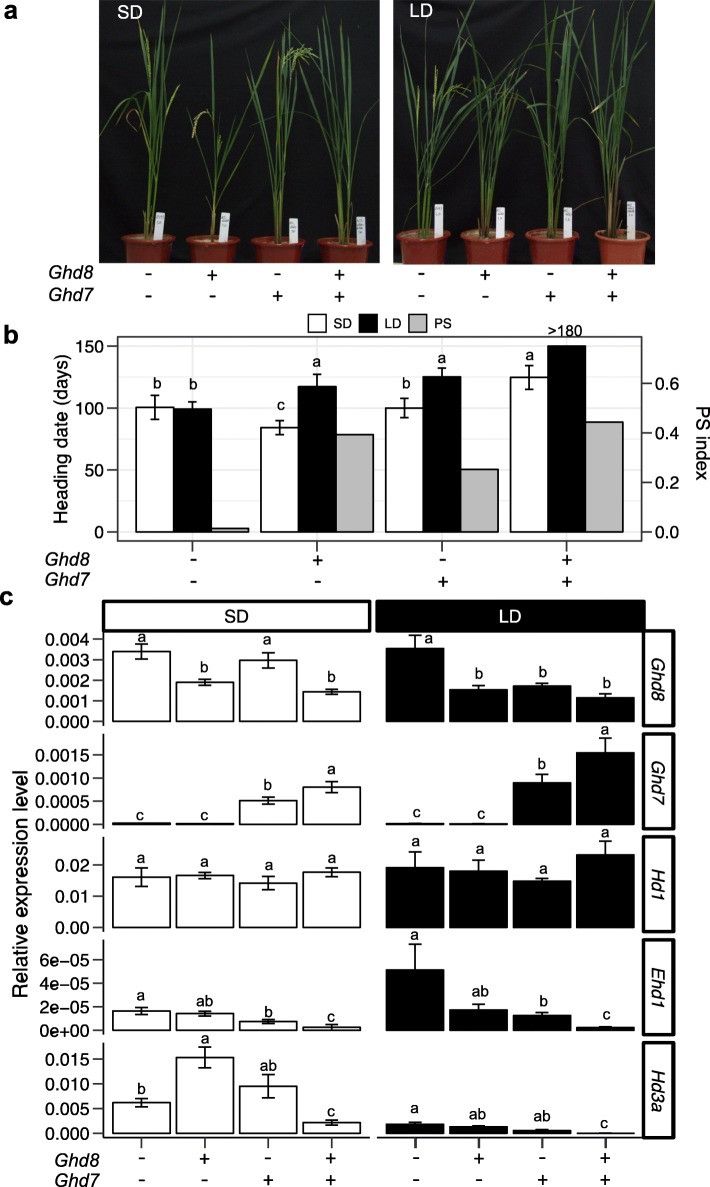
Expression levels of key flowering genes in the NILs with different combinations of *Ghd7* and *Ghd8* alleles. (a) Performance on the heading date of four NILs in the growth chamber under SD (10 h L: 14 h D) and LD conditions (14 h L: 10 h D). (b) Heading date of NIL (*ghd8ghd7*), NIL (*Ghd8ghd7*), NIL (*Ghd7ghd8*) and NIL (*Ghd8Ghd7*) under SD (white bars) and LD conditions (black bars), and photoperiod sensitivity (PS) index (gray bars). (c) Expression levels of *Ghd8*, *Ghd7*, *Ehd1*, *Hd3a* and *Hd1* under SD and LD conditions. The y-axis indicates the relative expression level of the gene normalized by *Ubiquitin*. Tukey’s HSD test was used to test the significance in heading date or expression among the four haplotypes under SD or LD conditions. The different letters above the bars denote the significance at *p* < 0.05. Mean ± SE (*n* = 4–6)

To explore the molecular basis of the interaction, transcription levels of five flowering-related genes (*Ghd8*, *Ghd7*, *Hd1*, *Ehd1* and *Hd3a*) were determined in 35-day-old seedling leaves of four NILs (NIL (*ghd8ghd7*), NIL (*Ghd8ghd7*), NIL (*ghd8Ghd7*), NIL (*Ghd8Ghd7*)) (Fig. 3a). NIL (*ghd7ghd8*) flowered at a similar time under both controlled SD (14 h dark: 10 h light) and LD conditions (10 h dark: 14 h light), showing insensitivity (PS index = 0.01) to the photoperiod change (Fig. 3b), although it carries the functional *Hd1* alleles. NIL (*Ghd8Ghd7*) significantly delayed HD compared with the other three NILs (*ghd8ghd7*, *Ghd8ghd7*, and *ghd8Ghd7*) under both SD and LD conditions and displayed the highest PS (PS index = 0.44). In particular, NIL (*Ghd8Ghd7*) did not flower, even after 180 d under LD conditions (Fig. 3b). Compared with NIL (*ghd8ghd7*), NIL (*ghd8Ghd7*) showed no significant difference in HD under SD conditions but significantly delayed HD under LD conditions. In contrast, NIL (*Ghd8ghd7*) delayed HD under LD conditions while promoting HD under SD conditions, which resulted in higher PS than NIL (*ghd8Ghd7*). Relative expression analyses of the five flowering genes confirmed that the transcription level of *Ghd7* was barely detected in NIL (*ghd8ghd7*) and NIL (*Ghd8ghd7*) due to the deletion of the *Ghd7* gene fragment in ZS97 [11]. However, *Ghd7* in NIL (*Ghd8Ghd7*) was expressed at a significantly higher level than that in NIL (*ghd8Ghd7*) under both SD and LD conditions (Fig. 3c). In addition, *Ehd1* and *Hd3a* expression levels in NIL (*Ghd8Ghd7*) showed a serious inhibition compared with that in the other NILs, which was in line with a severe delay in flowering time in NIL (*Ghd8Ghd7*) (Fig. 3b). The relative expression level of *Hd1* showed no significant difference among the four NILs under both SD and LD conditions, indicating that *Ghd8* and *Ghd7* did not affect the transcription level of *Hd1*. These results suggest that *Ghd8* might play a role in activating the transcription of the floral repressor *Ghd7* to suppress the expression of the downstream genes *Ehd1* and *Hd3a* under both LD and SD conditions, leading to late flowering.

### GHD8 mediated by HD1 binding to the promoter of *Ghd7*

**Fig. 4:**
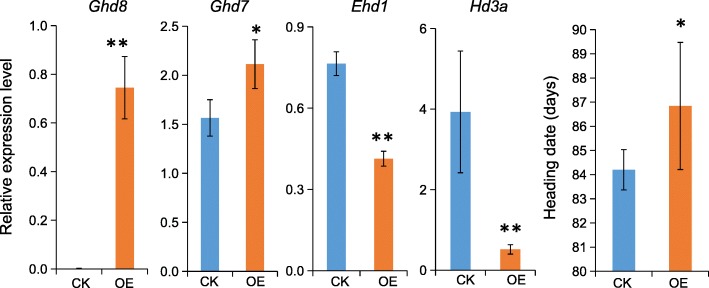
qRT-PCR analysis of several flowering-related genes in the *Ghd8* overexpression line. Leaves were harvested from 35-day-old seedlings of the Ubi::GHD8:GFP overexpression line (OE) and its corresponding negative control (CK) grown under natural LD conditions. Single or double asterisks denote significant differences at *P* < 0.05 or *P* < 0.01 by *t*-test

**Fig. 5:**
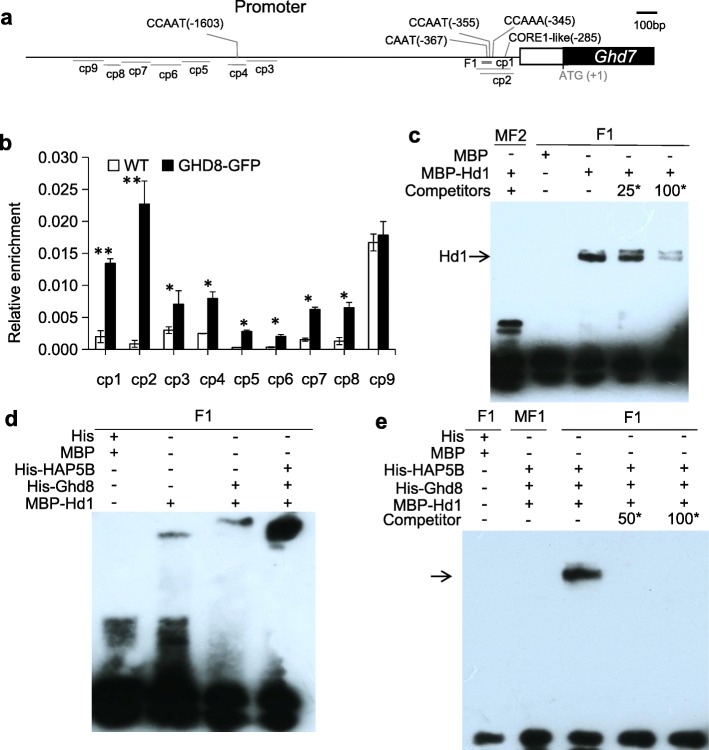
GHD8 binds to the promoter of *Ghd7*. **a** A schematic diagram of the *Ghd7* gene model showing the locations of two CCAAT-box motifs and one CORE1-like motif from the start codon ATG. The relative positions of the fragments from cp1 to cp9 used for the ChIP assay are given below the gene model; F1 indicates the fragment covering the CCAAT-box motif for the gel shift assays. **b** ChIP analysis of GHD8-GFP fusion protein using an anti-GFP antibody. The precipitated chromatin fragments were analyzed using the 9 primer sets for the target regions (cp1 to cp9) shown in (**a**). **c** Gel shift assays. Recombinant HD1 protein was incubated with biotin-F1 in the absence or presence of 25- or 100-fold molar excess of the unlabeled probe as the competitor. The arrow indicates the shifted band of HD1 protein. Maltose-binding protein (MBP) and His (polyhistidine)-tag protein were used as a negative control. **d** GHD8 interacts HD1 to binding F1 probe. **e** Interaction of the complex GHD8-OsHAP5b-HD1 (as shown by arrow) and F1 probe in the absence or presence of 50- or 100-fold molar excess of the unlabeled probe. The mutant F1 (MF1 or MF2) probe lacking the CCAAT box as a negative control

To understand how *Ghd8* interacts with *Ghd7* at the molecular level, we first conducted yeast two-hybrid assays for the protein interaction between GHD8 and GHD7. However, the assays did not reveal a direct interaction between them (Additional file 4: Figure S2a). It has been suggested that either Nuclear Factor A (NF-YA) could interact with NF-YB/NF-YC to form the NF-Y trimeric complex involved in transcriptional activation by binding specific *cis*-regulatory elements, such as the CCAAT-box or the CORE element [6, 32, 33]. To address whether *Ghd8* (NF-YB) regulates the expression of *Ghd7* by directly or indirectly binding the specific DNA region at the promoter of *Ghd7*, we investigated the CCAAT box and CORE element within the 2.5-kb promoter region of *Ghd7* by using the online promoter database PLACE [34]. The *Ghd7* promoter region contains two CCAAT-box motifs at the proximal and distal regions: − 355 bp and − 1603 bp upstream of the start codon (ATG) and one CORE1-like motif CATCCACA/TGTGGATG detected at − 285 bp [35, 36]. We then developed GHD8-GFP transgenic plants and conducted ChIP assays with extracts from these transgenic lines. The overexpression GHD8-GFP transgenic line showed a significant difference in HD with an approximately 3-d delay compared with the wild type under natural LD conditions (Fig. 4), indicating that the GHD8-GFP protein had biological function. The precipitated products in the presence of the antibody GFP and input (no antibody) were analyzed by qPCR using a set of 9 pairwise primers (Fig. 5a, b) corresponding to different regions in the promoter region. The ChIP assays showed that the adjacent fragments cp1 and cp2 covering the CCAAT-box at − 355 bp and CORE1-like motif at − 285 bp upstream of ATG displayed the highest enrichment in GHD8-GFP relative to that in the input control among all of the primer sets (Fig. 5b). These results indicate that the GHD8 protein might be involved in binding the CCAAT-box and/or CORE-like motif in the *Ghd7* promoter to activate its transcription.

We also found that GHD8 directly interacts with OsHAP5b in both yeast two-hybrid assays and pull-down experiments (Additional file 4: Figure S2 b and c). The genetic interaction study revealed that functional *Ghd8* and *Ghd7* are essential for *Hd1* controlling PS in rice (Fig. 2, Additional file 3: Figure S1), implying these three genes may work together to regulate flowering time. To test if the complex formed by GHD8 and OsHAP5b recognizes the CCAAT-box at the promoter of *Ghd7* by interacting with HD1, EMSA was performed by using the F1 probe that overlaps with the cp1 and cp2 fragments (Fig. 5a, c). The EMSA results revealed that the recombinant HD1 (MBP-HD1) alone could directly bind to the F1 fragment in vitro with a weak binding ability, and the excess unlabeled F1 fragment (competitor) inhibited the binding (Fig. 5c, d). We found that MBP-HD1 incubated either with His-GHD8 or with His-GHD8 and His-HAP5b could bind to the F1 fragment with different affinities in the EMSA assay (Fig. 5d). Moreover, a considerably stronger binding signal was observed when GHD8, HD1 and OsHAP5b proteins were incubated together with probe F1 (Fig. 5d), which is consistent with a previous study demonstrating that HD1 interacts with GHD8 and OsHAP5b to form a trimeric complex to bind the CORE motif in rice [30]. This result was also confirmed by using the competition assays with the unlabeled F1 fragment and the probe MF1 (a mutated F1 probe lacking the CCAAT element) (Fig. 5e).

## Discussion

### Molecular interaction of *Ghd8* and *Ghd7*

*Ghd7* and *Ghd8* have been reported as the major genes determining flowering time and plant growth [11]. In the present study, we demonstrate that *Ghd8* genetically interacts with *Ghd7*, delaying heading date in near-isogenic lines and in the rice core collection. Importantly, we found that the transcript level of *Ghd7* was activated by *Ghd8* through the GHD8-HAP5b-HD1 complex.

**Fig. 6:**
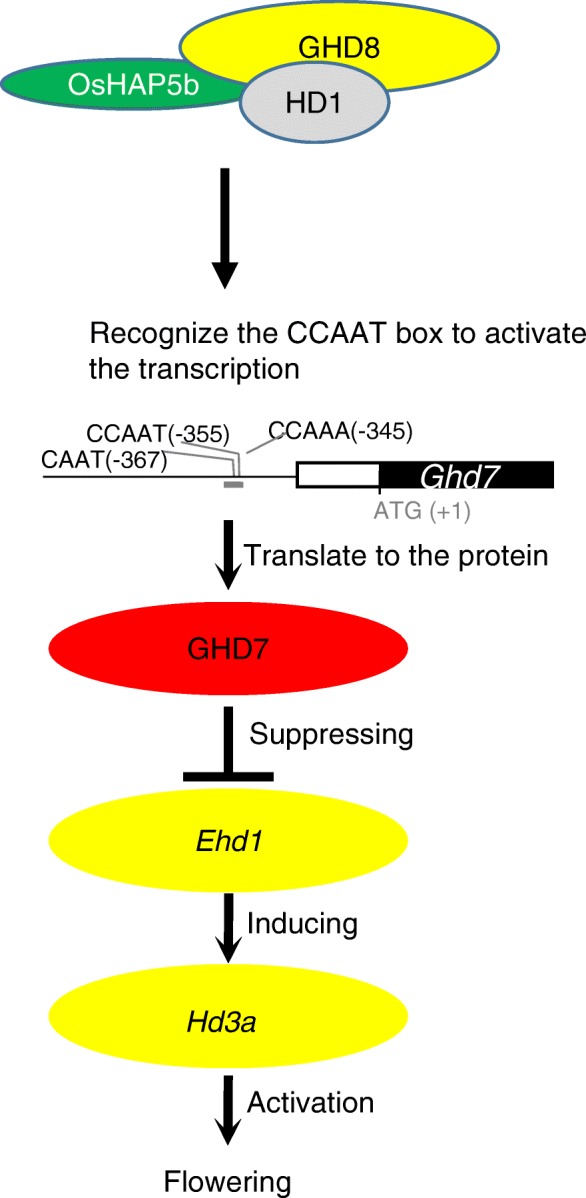
Model of GHD8 complex regulating flowering time. The heterotrimer GHD8-OsHAP5b-HD1 targets the CCAAT-box element and/or CORE motif of the *Ghd7* promoter to activate its expression, leading to suppression of *Ehd1* and downregulation of *Hd3a* expression

Under LD conditions, *Ghd7* strongly represses the expression of *Ehd1,* leading to decreased expression of florigen *Hd3a* to inhibit flowering in rice [11]. Recent studies have revealed that *Ghd7* is controlled at the transcriptional and posttranslational levels by other flowering time genes, such as *OsELF3* and *Hd16* [29, 37], which suggest that modification of *Ghd7* is a critical role in the decision of flowering time in the long-day repression pathway. A regulatory pathway in a previous report showed that *Hd1* upregulated the expression level of *Ghd7*, leading to downregulation of *Ehd1* and *Hd3a* under LD conditions [24]. Additionally, Nemoto et al. reported that the interaction between HD1 and GHD7 proteins resulted in a synergistic repression of *Ehd1* to cause a serious delay of flowering time [25]. The GHD7-HD1 complex represses *Ehd1* by binding to a *cis*-regulatory region of *Ehd1* [6], which suggested that *Hd1* was integrated into the LD repression pathway. Due to the high expression of *Ghd7*, *Ehd1* and *Hd3a* were repressed to delay flowering [27, 30]. Hence, *Ghd7* not only works as a signal integrator to receive the output from circadian clock [37], but also plays a role in transmitting regulation signals of other flowering-related genes, such as *Hd1* to *Ehd1*, to affect flowering time. It is notable that no difference in the *Hd3a* expression level was revealed in the NILs *ghd8Ghd7* and *ghd8ghd7*; intriguingly, *Hd3a* was repressed in NIL (*Ghd8Ghd7*) by approximately eight-fold compared with that in NIL (*Ghd8ghd7*) under SD conditions (Fig. 3c). These results suggest that *Ghd7* did play a role as a repressor of the expression of *Hd3a* in a dependent manner of functional *Ghd8* (Fig. 6). Furthermore, our data revealed the enhanced effect of *Ghd8* and *Ghd7* interaction on flowering time in the genetic background of ZS97 with a functional *Hd1*; however, the transcription level of *Hd1* remains unchanged in the NILs with the ZS97 background. This finding suggests that *Ghd8* might integrate the Hd1-Ghd7 pathway in the regulation of rice flowering through a posttranscriptional mechanism that the protein GHD8 or GHD7 might interact with HD1.

Previously, *Ghd8* was shown to play a similar role as *Hd1* with repression or activation in flowering time under LD or SD conditions [27, 30]. *Hd1* repressed *Hd3a* under LD conditions or activated gene expression under SD conditions in the presence of the nonfunctional *ghd7* [25]. In the current study, the dual function for *Ghd8* on flowering was also found to be dependent on the presence of the nonfunctional *ghd7* (Fig. 3b, c). In the comparison between NIL (*ghd8ghd7*) and NIL (*Ghd8ghd7*) with the functional *Hd1* background, *Ghd8* caused early flowering under SD conditions. Consistently, the *Hd3a* expression was much more activated in NIL (*Ghd8ghd7*) than that in NIL (*ghd8ghd7*). Previous study also revealed that under SD conditions, *Hd1* could activate the expression of *Ehd1* and *Hd3a* regardless of *Ghd7* functions [11]. In addition, it was reported that HD1-containing NF-Y complex has the capacity to bind a conserved response element in the *Hd3a* promoter [6]. These results suggest that *Ghd8* acts as an activator and might work together with *Hd1* to regulate *Hd3a* under SD conditions (Fig. 3). As mentioned above, the GHD7-HD1 complex could bind the *Ehd1* promoter under LD conditions [25]. The GHD8-HD1 complex promoted the flowering time through regulating *Hd3a* expression [30]. And GHD8-HAP5b-HD1 complex may activate *Ghd7* expression to enhance its repression effect on flowering time. These data point to the flowering regulatory pathway linking Ghd8 to Hd1 and Ghd7. Taken together, these results indicate that *Hd1* not only plays an important role in activating transcription under SD conditions but also incorporates GHD7 protein, inhibiting the expression of *Ehd1* in the LD repression pathway. Moreover, *Ghd8* could enhance the inhibitory role of *Ghd7* in flowering time under both SD and LD conditions (Fig. 3c). These findings suggest that the *Hd1* function become flexible to affect flowering time in terms of the photoperiod change through interacting with other transcription factors like *Ghd8* and *Ghd7.*

### GHD8-OsHAP5b-HD1 binds the CCAAT-box

*Ghd8* encodes a homolog of HAP3 or NF-YB subunit, together with HAP2 and HAP5 or NF-YA and NF-YC, constitutes the trimeric complex to bind the CCAAT-box at the promoter to activate the gene transcription [33]. However, in the absence of NF-YA, other transcription factors, such as CO or bZIP, also interact with NF-YB and NF-YC to form new complexes, leading to different binding sites that do not strictly bind to the CCAAT-box [30, 38–41]. For example, the CCT domain of CO and CO-like protein could interact with the complex of NF-YB and NF-YC to bind the CORE-element in *Arabidopsis* [35, 42]. In rice, the GHD8-OsHAP5b-HD1 complex has been reported to directly bind the CORE1 element on the promoter of *Hd3a* to regulate flowering [30]. In the current study, GHD8-OsHAP5b was found to interact with HD1 to form a trimeric complex to bind the CCAAT-box at the − 355 bp site of the *Ghd7* promoter. HD1 alone or the HD1-Ghd8 dimer were able to directly bind to the DNA fragment F1 that harbors the CCAAT-box, but this fragment did not cover the CORE1-like motif sequences (Fig. 5). Therefore, we propose that GHD8-HD1 or HD1 protein can recognize the CCAAT motif at the − 355 bp site.

In addition, the trimeric complex of NF-YB-NF-YC-CO appears more important to the binding of the CORE motif in *Arabidopsis* [35], and even mutation of any one of them can completely eliminate the binding ability of the complex. In contrast, the NF-YB1 subunit alone was reported to directly bind downstream genes without the assistance of other NF-Ys in the endosperm in rice [40, 43]. Our study revealed the low binding affinity of HD1 and HD1-GHD8 dimer on the CCAAT-box motif, suggesting that HD1 protein might be able to bind both the CCAAT-box motif and the CORE1 motif and have an overlapping function with OsHAP2/NF-YA and compete with these subunits to recognize the CCAAT-box motif [44]. Although the role of the CORE1-like motif in the binding stability of the GHD8-OsHAP5b-HD1 complex needs to be further investigated, the molecular interaction between *Ghd8* and *Ghd7* to control of *Hd3a* expression supports that the GHD8-OsHAP5b-HD1 regulatory complex is involved in the *Ghd7* repression pathway. These results suggest the existence of multiple targets for the GHD8-OsHAP5b-HD1 complex that fine tune the spatio-temporal regulation of gene expression in the flowering network, which might have a greatly contributed to the adaption of rice to various cultivation areas.

### Implications of *Ghd7* and *Ghd8* interaction

Asian cultivated rice has two subspecies: *indica* and *japonica,* both of which are distributed in different eco-geographic environments [31]. In the present study, *Ghd8* and *Ghd7*, as well as their interaction effect, were detected in response to the specific photoperiod conditions. The varieties with both weak functional *Ghd7* and *Ghd8* alleles dominate in the northern region between 35°N and 45°N, where farmers usually practice single planting in the summer cultivation season of long day-length (Additional file 5: Figure S3). The majority of varieties planted there are *japonica* subspecies [11, 27] and show a relatively low PS (average PS index = 0.2) (Additional file 1: Table S1). In parallel, the varieties with strong functional *Ghd7* and *Ghd8* with a relatively high PS are mainly found in subtropical and tropical regions between 25°N and 15°N. Additionally, a portion of varieties that contain both nonfunctional alleles *ghd7* and *ghd8* occur predominantly along 25°N latitudes (Additional file 5: Figure S3). This finding suggests that nonfunctional *ghd7* and *ghd8* are beneficial for the rapid growth of varieties required in this zone, where multiple plantings occur.

Notably, almost all of the northern *japonica* varieties carrying both functional alleles of *Ghd7* and *Ghd8* have functional *Hd1*(Additional file 5: Figure S3). However, these varieties flowered early (68–85 d) in natural LD conditions. This unexpected observation is different from the late heading in the NILs with the *indica* background, although they carry all functional alleles of *Ghd7*, *Ghd8* and *Hd1*. One explanation is that the *japonica* varieties might have divergent *Ghd7*, *Ghd8* or *Hd1* alleles with a reduced/weaker function that probably facilitates *japonica* rice adaptation to the northern/high latitudes [11, 27, 31, 45, 46]. Our study indicates that a high disequilibrium linkage of *Ghd7* and *Ghd8* exists in *japonica* rice (*p* < 0.01), and *Ghd8* or *Ghd7* also occurs in linkage disequilibrium with *Hd1* and/or *Hd3a* in either *indica* or *japonica*. This high linkage relationship among these genes suggests that some preferable genes in the regulation network might be selected together for suitable flowering (Additional file 6: Figure S4). The other possible explanation is that some flowering gene interaction unidentified in rice helps to counteract severely delayed flowering that arose by functional *Ghd7*, *Ghd8* and *Hd1* [23, 29].

There is a significant association between flowering time and grain yield in rice. A longer flowering time is usually associated with higher grain yield. However, there is a trade-off between increased yield potential and delays in HD. Therefore, in terms of the action of *Ghd7* and *Ghd8*, which play a crucial role in the regulation of flowering, these genes should be considered together in high-yield breeding programs, especially for those varieties used in the low latitude regions, where multiple plantings are commonly applied for obtaining high yield per area. As rice is the main source of daily calories, deciphering the combined effects of *Ghd8*, *Ghd7*, and *Hd1* in flowering regulatory pathways will help elucidate the adaptation mechanism to various photoperiod conditions and will be beneficial in molecular design for the development of rice varieties with high yield potential.

## Conclusions

This study investigates the genetic interaction effect between two major genes, *Ghd8* and *Ghd7*, by using the segregating population generated from the cross between NIL lines and a natural germplasm panel in rice. The molecular basis of their interaction was further identified by using a series of experiments, including EMSA and ChIP. We found that *Ghd8* regulates transcript of *Ghd7* through the GHD8-OsHAP5b-HD1 regulatory complex. Thus *Ghd8* and *Ghd7* together with *Hd1* largely explain seriously delayed flowering time, increased plant height, and improved yield potential in rice varieties. We also found that both functional *Ghd8* and *Ghd7* are pivotal for rice PS controlled by *Hd1* and *Hd3a*, which is highly important for rice varieties to adapt to different environments. These findings provide insights into the molecular and genetic bases of the gene interaction on crop productivity and adaptation and reveal that the selectivity and combination of different functional alleles could be highly useful for improving yield potential in rice breeding programs.

## Methods

### Plant material and growth conditions

The panel of 196 rice accessions was used for nucleotide sequencing analysis [47, 48]. These accessions were obtained from China National Rice Research Institute and the International Rice Research Institutes. The varieties were grown in Wuhan (N30.52, E114.3) under natural LD conditions and in Hainan (N18.48, E110.02) under natural SD conditions for the measurements.

Two advanced backcross lines harboring the genomic regions with either functional *Ghd7*, *Ghd8* or *Hd1* were used to generate F_1_ hybrids [20]. The F_1_ plants were then backcrossed with ZS97 twice to create the BC_2_F_1_ generation. The segregating population of BC_2_F_2_ containing approximately 200 individuals was grown in Hainan. The SSR markers RM5436, PID2, and RM121 were used to select the heterozygous segments at *Ghd7*, *Ghd8* and *Hd1* during the crossing scheme. The four homozygous genotypes of *Ghd8Ghd7*, *Ghd8ghd7*, *ghd8Ghd7* and *ghd8ghd7* with the common background of ZS97 were selected as the near-isogenic lines (NILs) in which *Hd1* is functional.

To compare the HD and PH of the NILs under different light/dark conditions, 10 plants for each NIL were grown in LD (14 h light: 10 h dark) and SD (10 h light: 14 h dark) conditions in growth chambers. Flowering time was scored from 8 individuals per line. HD was defined as the time when the first panicle appeared from the flag leaf.

### DNA sequencing

The rice core collection was genotyped by sequencing at the coding region of *Ghd7*, *Hd1*, *Ehd1*, *Hd3a* and *Ghd8* covering the functional site described in recent papers [11, 12, 27]. The PCR amplified fragments were sequenced directly using BigDye Terminator Cycle Sequencing v3.1 (Applied Biosystems, Foster, USA) after digestion and purification according to the manufacturer’s specifications. The primers used are listed in Additional file 2: Table S2.

### Gene expression analyses

Leaves from the main culm of NILs of 35-day-old plants were harvested to analyze the transcription levels of *Ghd8*, *Ghd7*, *Hd3a*, *Ehd1* and *Hd1*. The NILs were grown in the growth chambers respectively under SD and LD conditions at 30 °C in the light and 26 °C in the dark. Samples were collected at Zeitgeber time (ZT) 4 under SD conditions and at ZT 8 under LD for gene expression analysis. ZT 0 indicates dawn. The samples were collected for RNA extraction using an RNA extraction kit (TRIzol Reagent, Invitrogen). The time points for sampling corresponded to the expression peak for each gene according to the reports [11, 27]. Approximately 2 μl of RNA was used to synthesize the cDNA in a 20 μl reaction volume by using SuperScriptII reverse transcriptase (Invitrogen) after treatment with DNase I to remove the contaminating genomic DNA. Real-time PCR (RT-PCR) was performed in a total volume of 25 μl on an Applied Biosystems 7500 Real-Time PCR System, which contained 2 μl cDNA, 2 μl primers (0.4 μM), 8.5 μl distilled water and 12.5 μl of SYBR® Green PCR Master Mix (Applied Biosystems) for each reaction. The expression data were normalized by using the *ubiquitin* gene with the relative quantification method [49]. All experiments were conducted in at least three biological and three technical replicates. The primers used for the transcription analyses are listed in Additional file 2: Table S2.

### Yeast two-hybrid assays

The protein-coding regions of *Ghd7*, *Ghd8* and *OsHAP5b* were amplified using gene-specific primers with added restriction enzyme sites, respectively (Additional file 2: Table S2). Then, the *Ghd7* or *OsHAP5b* product amplified by PCR was fused into the activation domain (AD) vector pGADT7 as a prey system with *EcoR*I and *Xho*I sites, and the *Ghd8* product with *EcoR*I and *BamH*I sites was fused to the DNA-binding domain (BD) vector pGBKT7 as bait system. All constructs were verified by sequencing. The cotransformation of two plasmids carrying the *Ghd8*, *OsHAP5b* and *Ghd7* genes into the AH109 yeast strain and cell culture were performed according to the manufacturer’s protocols (Matchmaker Gold Yeast Two-Hybrid System, Clontech). Constructs of pGBKT7–53 (pBD-53) and pGADT7-T (pAD-T) served as positive controls, and constructs of pGBKT7-Lam and pGADT7-T (pAD-T) served as negative controls.

### In vitro pull-down assay

The coding region of *Ghd8* was cloned into the pET-32a vector (Novagen) and pGEX-6P-1 vector (GE Healthcare) with *EcoR*I and *Xho*I sites, respectively. To obtain the OsHAP5b protein, we used the same method for GHD8. The recombinant expression vector was expressed in *Escherichia coli* Transetta (DE3) cells (Transgen). The pull-down experiment was performed as described previously [50]. In brief, supernatants with equal amounts of Glutathione S-transferase (GST) or OsHAP5b-GST with GHD8-His recombinant proteins were incubated for 6 h at 4 **°**C in a total volume of 2 ml of pull-down buffer (20 mmol Tris-HCl, pH 8.0, 200 mmol NaCl, 1 mmol EDTA, 0.5% Lgepal CA-630 and protease inhibitor), after which 200 μl of GST resin was added (GE Healthcare; 17–5132-01) and the mixture incubated for 2 h at 4 **°**C. The binding reaction was then washed 5 times (10 min each time at 4 **°**C) using the pull-down buffer. After extensive washing, the pulled-down proteins were eluted by boiling at 95 **°**C for 10 min, separated on 12% SDS-PAGE and detected by immunoblots using an anti-GST antibody (Abcam; ab19256) and anti-His antibody (Abcam; ab9108), respectively.

### ChIP (chromatin immunoprecipitation)

For ChIP assays, wild-type and GHD8-GFP transgenic lines were used for chromatin extraction and immunoprecipitation following the method described [51]. Briefly, young leaves from approximately 35-day-old seedlings were collected under LD conditions (15 h light: 9 h dark). After treated with formaldehyde to cross-link the proteins to the DNA, the cross-linked lysate was sonicated to shear DNA to the smaller fragment size. The soluble chromatin fragments were isolated and purified for the following steps. Immunoprecipitation with anti-GFP (Abcam; ab290) was performed for wild-type and GHD8-GFP transgenic lines with 3 repeats. The precipitated DNA was analyzed by quantitative RT-PCR using specific primer sets listed in Additional file 2: Table S2, designed to cover the CCAAT-box element within a 2-kb primer region upstream of ATG of *Ghd7*.

To construct the GHD8-GFP fusion, maize (*Zea mays*) *ubiquitin* promoter-GFP cassette from pU1301 was used to substitute the GUS fragment of pCAMBIA1391Xb to create a modified pCAMBIA1391Xb [51]. The conservative domain within the *Ghd8* coding region involving in the binding and interaction function was amplified and inserted into the *Kpn*I and *BamH*I sites of the modified pCAMBIA1391Xb to obtain the construct Ubi::GHD8-GFP for rice transformation. The *Agrobacterium* mediated genetic transformation was used to generate the transgenic plants with Ubi::GHD8-GFP construct in the Nipponbare background.

### Electrophoretic mobility shift assay (EMSA)

The pET32-based expression vectors for GHD8 and OsHAP5b were used to express recombinant proteins fused to a thioredoxin and a His (polyhistidine)-tag in tandem at their N-terminal ends. An *E. coli* BL21 strain (Transgen) was transformed with vectors and grown at 37 °C. pMAL vector (New England Biolabs, E8000S) was used to obtain the recombinant HD1 proteins with the maltose-binding protein (MBP) at the down-stream. The recombinant proteins pET32-GHD8, pET32-OsHAP5b and pMAL-c2X-HD1 were purified with Ni-NTA agarose (QIAGEN; No. 30210) and amylose resin beads (New England Biolabs) respectively. The *Ghd7* promoter fragment F1 (including the putative binding site CCAAT-box) were produced by annealing of 3′-biotin-labeled oligonucleotides Ghd7F/R (Sangon Biotech), respectively. DNA binding reactions were performed in the presence or absence of unlabeled F1 and labeled mutated F1 (MF1 and MF2) fragments at room temperature for 20 min in 5 mM Tris, pH 7.5, 25 mM KCl, 0.5 mM DTT, 5 mM MgCl_2_, 2.5% glycerol, 0.05% NP-40 and 50 ng/uL poly (dI-dC). We followed the protocol from the LightShift Chemiluminescent EMSA Kit (Thermo; No.20148), and the samples were run on 5% polyacrylamide gels. The primers used for EMSA are listed in Additional file 2: Table S2.

### Linkage disequilibrium analysis

Linkage disequilibrium analysis of *Ghd7*, *Ghd8*, *OsHAP5b*, *Hd1* and *Hd3a* was performed using the software TASSEL 3.0.147 (http://www.maizegenetics.net/). The sequences including single nucleotide polymorphisms in the promoter region (1.5 kb upstream of ATG), coding region and 3’UTR (1 kb downstream of the stop codon) of these genes in 532 varieties were obtained from the website (http://ricevarmap.ncpgr.cn/django/snp_id/). All SNPs in each gene from the varieties were input into TASSEL to conduct linkage disequilibrium analysis. Out of 532 varieties, 2 groups, each including 295 *indica* and 156 *japonica* were also separated for linkage disequilibrium analysis, and a threshold of 1% was set to filter the SNPs with low frequencies.

### Statistical analysis

Analysis of variance (ANOVA) of gene interaction on the assayed traits in the F_2_ segregating population derived from the cross of NIL (*Ghd7*) × NIL (*Ghd8*) was conducted using Statistica software [52]. Tukey’s HSD (honest significant difference) test in JMP software was employed to determine which groups were significantly different [53].

## Supplementary information

**Additional file 1. Table S1.** Functional alleles of *Ghd8*, *Ghd7* and *Hd1* and promoter type of *Hd3a* in the panel of 196 rice varieties.

**Additional file 2. Table S2.** PCR primers and EMSA probes used in this study.

**Additional file 3. Figure S1.** Heading date (HD) in the genetic population segregated at *Ghd8*, *Hd1* and *Ghd7*. *Ghd8*, *Hd1* and *Ghd7* indicate the functional alleles, and lowercase *ghd8*, *hd1* and *ghd7* indicate the nonfunctional alleles. *Ghd8/ghd8*, *Ghd7/ghd7* and *Hd1/hd1* indicate heterozygote.

**Additional file 4. Figure S2.** Detection of protein interaction. (a) Yeast two-hybrid assays for GHD8 and GHD7 interaction. (b) Yeast two-hybrid assays for GHD8 and OsHAP5b. (c) Pull-down assay for GHD8 and OsHAP5b in vitro.

**Additional file 5. Figure S3.** Geographic distribution of 196 rice varieties. All 196 rice varieties were highlighted by four combinations of *Ghd8* and *Ghd7* and subdivided into *indica* or *japonica* subspecies. The stacked bar graph indicates the distribution and frequency of four combinations (haplotypes) of *Ghd7* and *Ghd8* alleles. The map image was taken from Wikimedia Commons: https://commons.m.wikimedia.org/wiki/File.

**Additional file 6. Figure S4.** Linkage disequilibrium patterns among *Ghd8* and *Ghd7* and the other related flowering genes *Hd1*, *Hd3a* and *OsHAP5b* in 532 rice varieties. Total population, indica and japonica subgroups individually were used for the analysis.

## Data Availability

All the supporting data are included within the article. The other dataset used and/or analyzed during the current study not included here are available from the corresponding author on request.

## Abbreviations

ChIP: Chromatin immunoprecipitation
EMSA: Electrophoretic mobility shift assay
HAP: Heme activator protein
HD: Heading date
LD: Long-day conditions
NF-Y: Nuclear transcription factor Y
NIL: Near isogenic lines
PS: Photoperiod sensitivity
QTL: Quantitative trait locus
SD: Short-day conditions
