# Supplementary figures and images for: Ghd8 controls rice photoperiod sensitivity by forming a complex that interacts with Ghd7
Source: BMC Plant Biol. 2019 Nov 1;19:462. doi: 10.1186/s12870-019-2053-y (PMC6825352; doi:10.1186/s12870-019-2053-y)

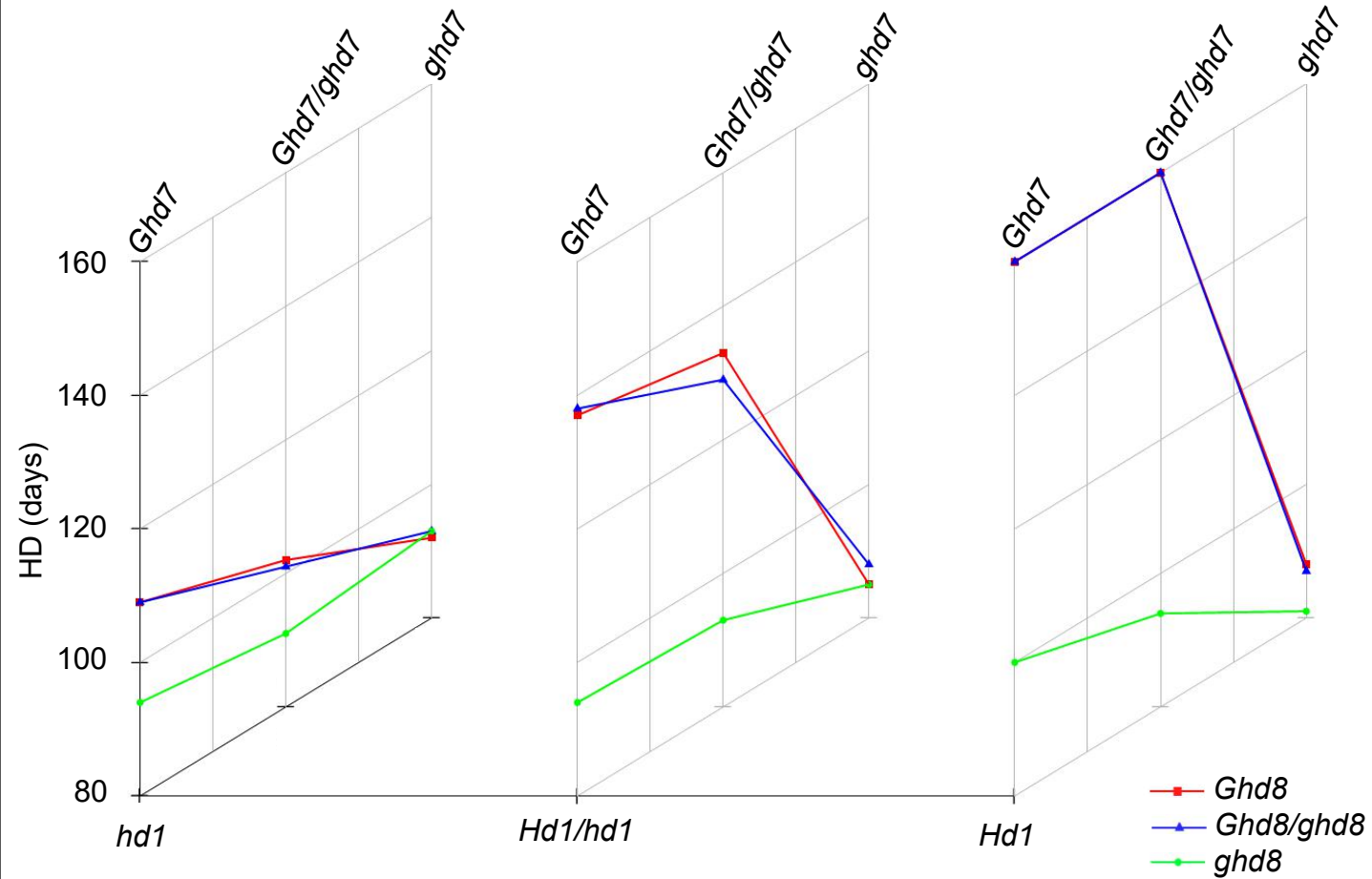

Supplement: Supplementary file 3 — Additional file 3. Figure S1. Heading date (HD) in the genetic population segregated at Ghd8, Hd1 and Ghd7. Ghd8, Hd1 and Ghd7 indicate the functional alleles, and lowercase ghd8, hd1 and ghd7 indicate the nonfunctional alleles. Ghd8/ghd8, Ghd7/ghd7 and Hd1/hd1 indicate heterozygote. [file 12870_2019_2053_MOESM3_ESM.pdf]

**a**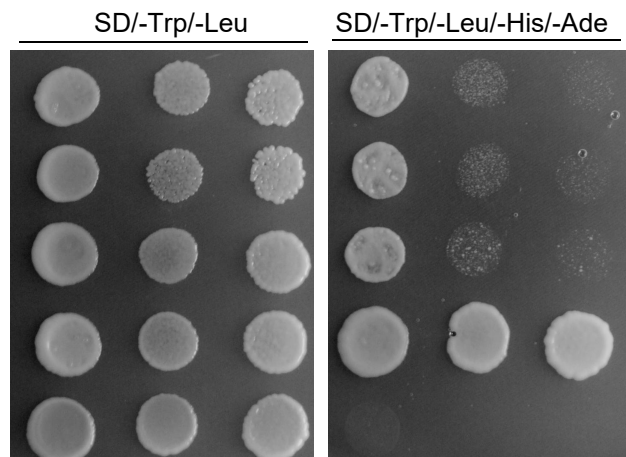

BDGHD8 / GHD7-AD

BDGHD8 / GHD7-AD

BDGHD8 / AD-empty vector

BK-53 / AD-T7 (Positive)

BK-Lam / AD-T7 (Negative)

**b**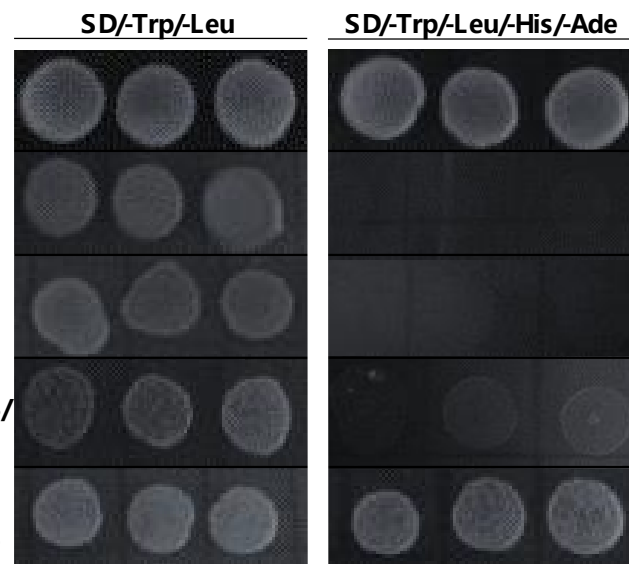**c**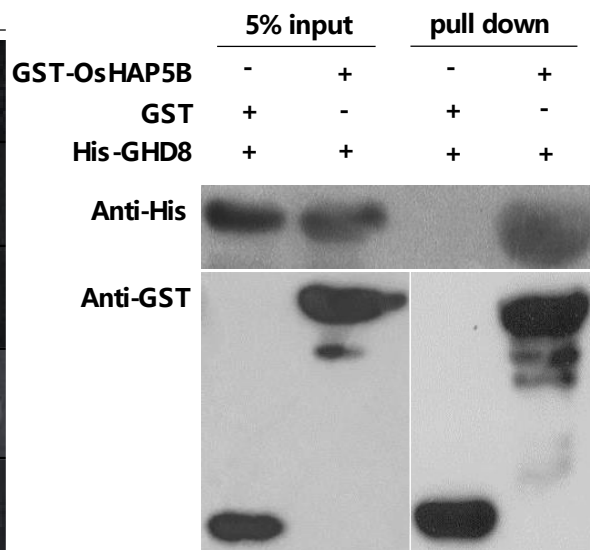

Supplement: Supplementary file 4 — Additional file 4. Figure S2. Detection of protein interaction. (a) Yeast two-hybrid assays for GHD8 and GHD7 interaction. (b) Yeast two-hybrid assays for GHD8 and OsHAP5b. (c) Pull-down assay for GHD8 and OsHAP5b in vitro. [file 12870_2019_2053_MOESM4_ESM.pdf]

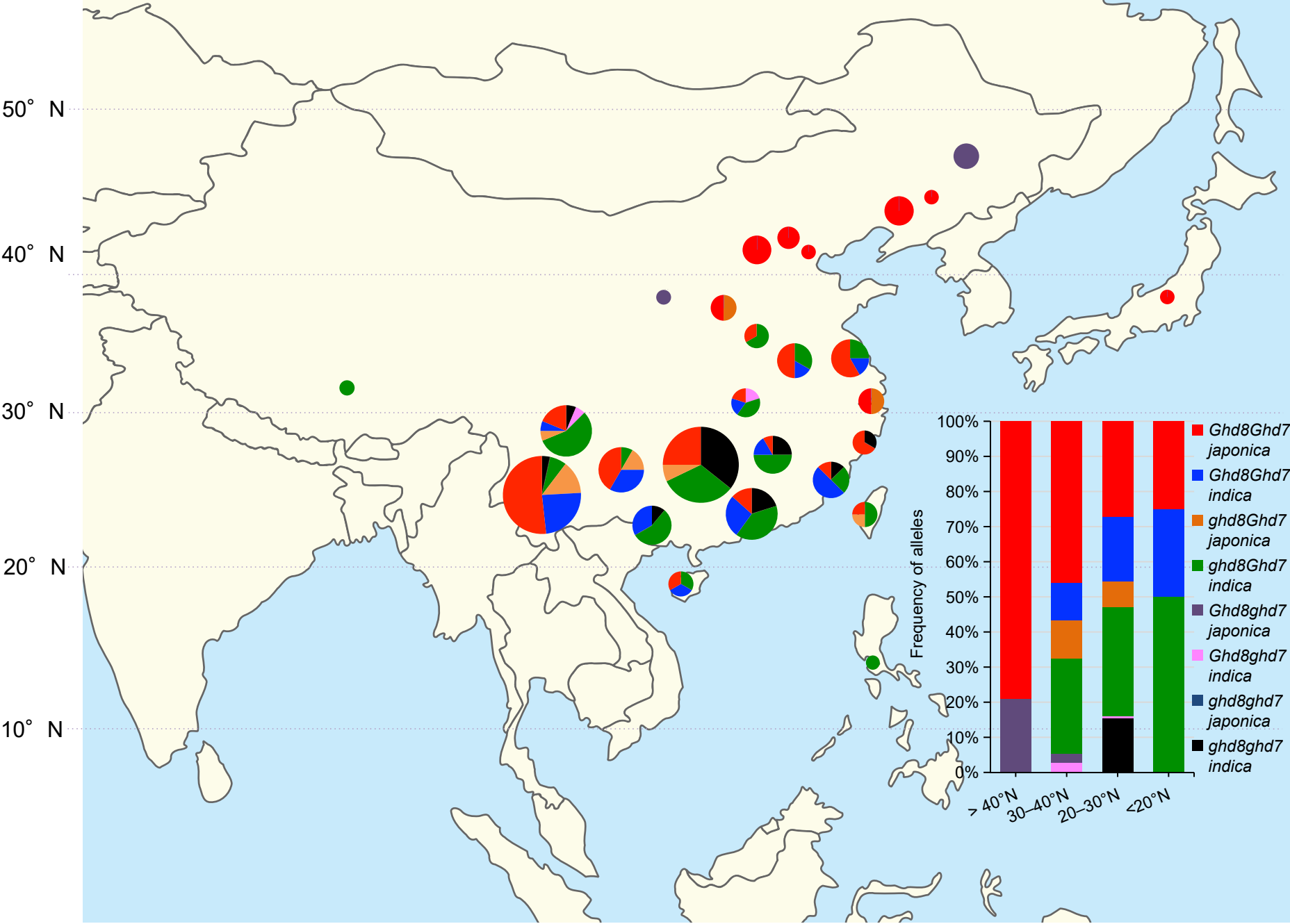

Supplement: Supplementary file 5 — Additional file 5. Figure S3. Geographic distribution of 196 rice varieties. All 196 rice varieties were highlighted by four combinations of Ghd8 and Ghd7 and subdivided into indica or japonica subspecies. The stacked bar graph indicates the distribution and frequency of four combinations (haplotypes) of Ghd7 and Ghd8 alleles. The map image was taken from Wikimedia Commons: https://commons.m.wikimedia.org/wiki/File. [file 12870_2019_2053_MOESM5_ESM.pdf]

# *indica + japonica*

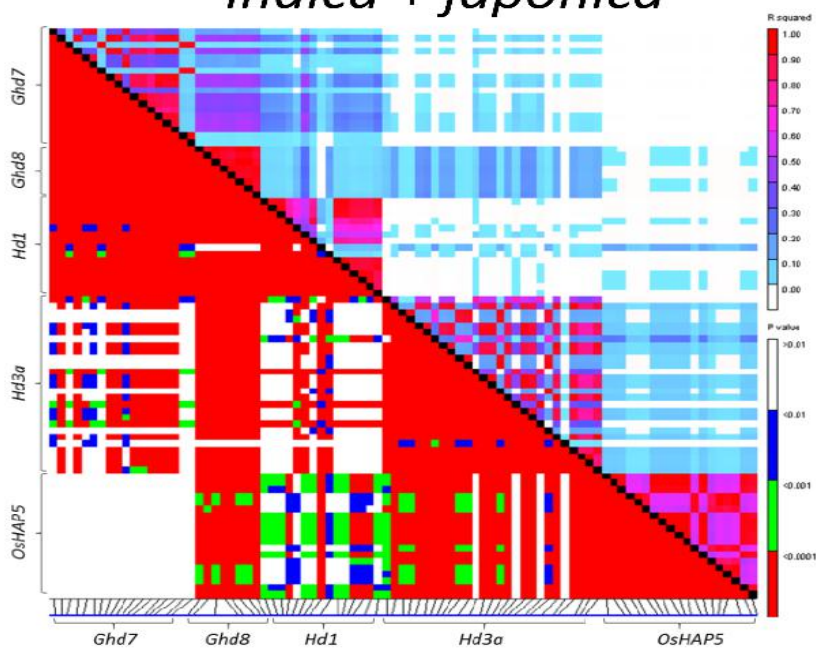

# *indica*

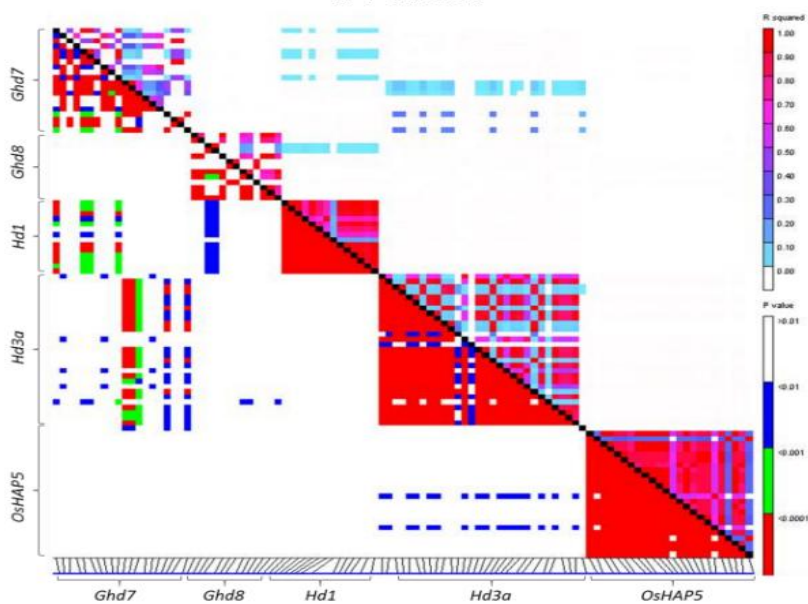

# *japonica*

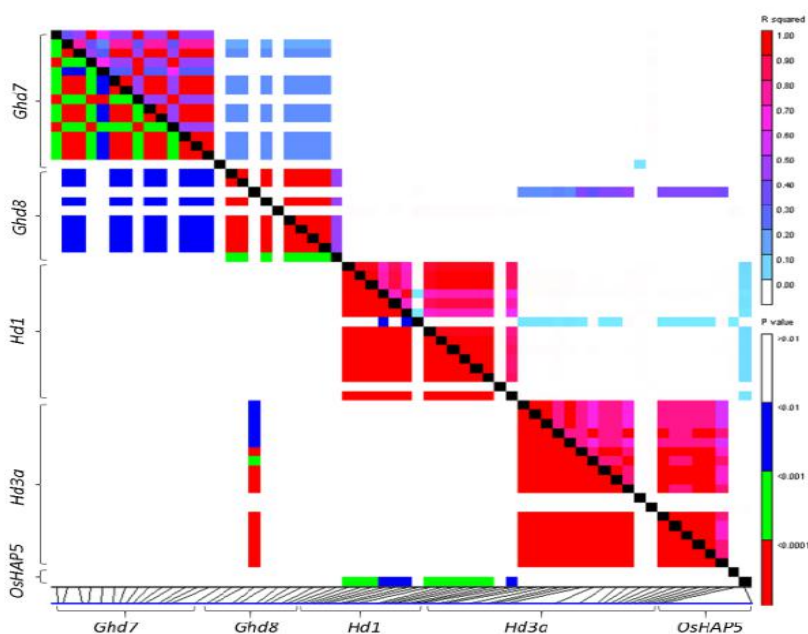

Supplement: Supplementary file 6 — Additional file 6. Figure S4. Linkage disequilibrium patterns among Ghd8 and Ghd7 and the other related flowering genes Hd1, Hd3a and OsHAP5b in 532 rice varieties. Total population, indica and japonica subgroups individually were used for the analysis. [file 12870_2019_2053_MOESM6_ESM.pdf]
